# Potential biomarkers for esophageal cancer

**DOI:** 10.1186/s40064-016-2119-3

**Published:** 2016-04-16

**Authors:** Cheng Tan, Xia Qian, Zhifeng Guan, Baixia Yang, Yangyang Ge, Feng Wang, Jing Cai

**Affiliations:** Department of Radiation Oncology, Nantong Tumor Hospital, Affiliated Tumor Hospital of Nantong University, Nantong, 226321 China

## Abstract

Esophageal cancer, which consist of esophageal adenocarcinoma and esophageal squamous cell carcinoma, is one of the most common malignant tumors in the world, especially in the south of Iran and China. To find and investigate the biomarkers in the initiation, development and progression of esophageal cancer will help us predict the prognosis of esophageal cancer patients and improve the curative effect and survival rate. Here, we reviewed the potential biomarkers of esophageal cancer in three aspects: Immunohistochemical markers, blood-based markers, miRNA markers and Gene expression profiling. All these biomarkers provided promising therapeutic targets for the diagnosis, treatment, and prognosis of esophageal cancer.

## Background

Esophageal carcinoma (EC) is one of the most common malignant tumors in the world, with a wide distribution in the south of Iran and China (Zali et al. 2011). It is a very deadly disease, with roughly 480,000 new patients every year, and it is the sixth leading cause of cancer deaths worldwide, the fifth in men and the eighth in women (Jemal et al. 2011). Around 80 % of the cases worldwide occur in less developed regions. In both sexes there are more than 20-fold differences in incidence between the different regions of the world, with rates ranging from 0.8 per 100,000 in Western Africa to 17.0 per 100,000 in Eastern Asia in men, and 0.2 per 100,000 in Micronesia/Polynesia to 7.8 per 100,000 in Eastern Africa in women.(Glbocan 2012: Estimated Cancer Incidence, Mortality and Prevalence worldwide in 2012. Available at http://globocan.iarc.fr). More than 50 % of EC patients presented with distant metastasis when they were first diagnosed (Enzinger and Mayer 2003). The most common type of EC is esophageal squamous cell carcinoma (ESCC), with increasing morbidity in Western countries. The other type is esophageal adenocarcinoma (EAC), which can occur in Barrett’s epithelial cells, as well as distal esophageal mucosa and cardia. The common symptoms of EC are dysphagia, chest pain, weight loss, pressure or breastbone burn feel, and cough. The risk factors for this malignancy include smoking, diet, gastroesophageal reflux disease (GERD), and human papillomavirus (HPV) (Salehi et al. 2013). EC detection methods include esophagoscopy, positron emission tomography Raman spectrum, immunohistochemistry (IHC), and biopsy. The last method is the diagnostic standard of esophageal cancer, and it is used to confirm the physical and imaging examination results (Cerfolio et al. 2005). Obtaining accurate pre-treatment staging and then subsequently providing stage-appropriate treatment is crucial in optimizing esophageal cancer outcomes. Currently, it is based in the cost-effective use of Endoscopy, Endoscopic ultrasound, CT and biopsy (Berry 2014).

The usual treatments for EC are surgery, radiotherapy, and chemotherapy, of which surgery is the most effective option. Despite the combination of modern surgical procedures, radiotherapy, and chemotherapy, local recurrence is common in patients in the advanced stages of the disease. The presence of regional lymph node metastasis in patients with tumor invasion and metastasis disqualifies them from surgery (Rice et al. 2009). The survival rate is significantly low, and the average 5-year survival rate of one-third of patients is 35.45 % (Thompson et al. 2008). These clinical facts clearly show that early detection is crucial to treatment. However, only 30 % of early patients can be examined (Rice et al. 1998). As a result, studies on the molecular mechanism of the invasion and metastasis of EC are vital. Biomarkers can be detected using different detection methods, such as blood testing, IHC, molecular pathology, gene expression profile and biopsy. The biomarkers of EC are valuable in predicting the outcome and guiding treatments of the disease. Here, we review biomarkers in EC detection, diagnosis, treatment, and prognosis.

### Immunohistochemical biomarkers

IHC is the most widely applied pathological technique in determining the expression of tumor-associated proteins and in studying the prognostic and clinical relevance of biomarkers. To date, numerous investigations have demonstrated that many immunohistochemical markers are potential diagnostic, prognostic, or predictive indicators of EC. The role of IHC in EC is important to elucidate pathways for epidermal growth, angiogenesis, and apoptosis. In this section, epidermal growth factor receptor (EGFR), p53, vascular endothelial growth factor (VEGF), and estrogen receptor (ER) will be described in detail.

EGFR is a transmembrane protein with intrinsic tyrosine kinase activity. It is associated with specific ligands, such as EGF and TGF-α, resulting in the homodimerization of EGFR or in heterodimerization with the other EGFR receptor family members. Elevated levels of EGFR or increased expression levels of the EGFR gene have been reported in a number of human cancers of epithelial origin, including head and neck, thyroid, breast, and colon cancers. In a subset of these cancers, most notably breast, colorectal, and esophageal cancers, increased EGFR expression is associated with advanced disease, tumor metastases, and poor prognosis (Wang et al. 2013). A clinical study was carried out in the University of Texas MD Anderson Cancer Center (Wang et al. 2007), wherein the expression of EGFR in EAC was examined. The results revealed that EGFR expression in EAC is closely related to the pathological grade and lymph node metastasis, as well as poor disease-free and overall survival. However, it is independent of T staging. Sudo et al. (2007) investigated the presence of EGFR mutations in 19 esophageal cancer cell lines and primary tumors by PCR and DNA sequencing targeting exons 18, 19, 20 and 21. They found three of the 19 cell lines had the same silent mutation at nucleotide 2607, a G-to-A substitution in exon 20. One of the 50 patients had an EGFR mutation in codon 719, resulting in an amino acid substitution from glycine to aspartic acid. These findings suggested that the expression of EGFR is associated with poor prognosis and can be used to predict patient outcome.

As a member of the EGFR family, epidermal growth factor receptor 2 (HER2) has become a therapeutic target for some tumors and has received much research attention. HER2, a transmembrane receptor tyrosine kinase family member, is involved in cell regulation, cell growth, survival, differentiation, and migration of important substances (Spector and Blackwell 2009). Its positive expression in breast cancer has been elucidated and applied to treatment and prognosis in clinical practice (Penault-Llorca et al. 2009). The research scope of HER2 expression is various cancer types, such as gastric cancer and esophageal cancer, is also expanding. The relationship between ESCC and HER2 has been investigated, but a definite conclusion has not been reached (Lesnikova et al. 2009). In a study involving 99 patients, the 7p12 (C-myc), 8q24.12 (EGFR), and 20q13.2 genes were mostly amplified in severe dysplasia and adenocarcinoma, suggesting that the correlation between ESCC and HER2 is not exact (Rygiel et al. 2007). In addition, a prospective clinical trial is currently underway. The primary results suggested that the sensitivity and specificity for diagnosing severe dysplasia or adenocarcinoma are 88 and 100 %, respectively, by detecting HER2, c-myc, 20q13.2, and aneuploidy (Pacha et al. 2012). In a recent meta-analysis (Chan et al. 2012), 1464 esophageal cancer patients in 14 studies were observed, with 322 (22 %) HER2-positive patients. The five-year mortality was significantly higher in the HER2-positive group [odds ratio (OR) 1.43, 95 % CI 1.04–1.95, P = 0.03] than that in the controls. In addition, positive correlations between five-year mortality and HER2-positive squamous cell carcinoma (OR 2.88, 95 % CI 1.34–6.17; P = 0.006) and adenocarcinoma (OR 1.91, 95 % CI 1.15–3.17; P = 0.01) were found. In the abovementioned studies, the survival rate of ESCC patients with HER2-positive expression decreased, possibly because of increased radiation resistance (Dreilich et al. 2006) and the use of cisplatin-based chemotherapy (Akamatsu et al. 2003).

A study on the expression of E-cadherin, α-catenin, and β-catenin in EAC revealed that the decrease in the expression of the three proteins is associated with a decrease in the survival of patients. The expression of E-cadherin in patients with neoadjuvant chemotherapy significantly decreased, with other traditional clinical and pathological features, including the depth of tumor invasion, lymph node metastasis, and clinical stage.

In addition, p53 was detected through IHC, and it was found to play a supplementary role in the diagnosis of dysplasia, particularly low-grade dysplasia. Although it is not approved for clinical use, p53 may be particularly useful for providing another basis for the diagnosis of dysplasia (Kaye et al. 2009). Patients with dysplasia are now advised to be examined using p53 IHC to improve diagnostic reproducibility (Fitzgerald et al. 2014). Furthermore, in some studies, EC patients with abnormal p53 IHC show three to eight times more rapid progression of this disease, indicating that it can also be used as a predictor of disease progression (Sikkema et al. 2009). Combined with other factors, p53 can be considered a risk factor for disease progression. For example, in the diagnosis of low-grade dysplasia, three pathological scientists agreed that the expression of p53 protein immunohistochemical abnormalities may be considered a major risk of disease progression (Skacel et al. 2002).

VEGF is a potent source of angiogenesis, and it is responsible for the development and maintenance of a vascular network that promotes tumor growth and metastasis for a wide range of human tumors and human cell lines (Weston et al. 2001). In clinical specimens, the expression of VEGF-C in EC tissues was significantly higher than that in noncancerous tissues (P = 0.026). Other studies have confirmed that the positive expression of VEGF-C may be closely related to the progression of the disease (Sikkema et al. 2009; Skacel et al. 2002; Weston et al. 2001; Bird-Lieberman et al. 2012).

In a recent study (Zuguchi et al. 2012), ER types ERa and ERb were detected in 90 cases of ESCC. ERa and ERb were detected in the nuclei of ESCC (41.1 and 97.8 %, respectively). Collecting relevant evidence may prove that ERb can be used as an indicator of a treatment or prognosis in the future.

In addition, MEKK3/MAP3K3 overexpression is important in the development of cancer (Kumar et al. 2007). Glickman et al. (2001) analyzed a series of EACs and squamous cell carcinoma and demonstrated p53 expression in 100 % of ESCC with no expression identified in EAC. DiMaio et al. (2012) examined the expression of eight immunohistochemical markers, including the novel markers SOX2 (sex determining region Y-box 2) and AGR2 (Anterior gradient 2), in a large series of EAC and ESCC to determine the optimal panel of immunohistochemical markers for distinguishing these tumors. In clinical and scientific studies, the molecular mechanism of many diseases has been revealed by IHC and molecular pathology, which is related to the prognosis of many diseases.

### Blood-based biomarkers

In the early stages of carcinogenesis, the antigen immune response is believed to occur during cancer immune surveillance, in which the antigen is recognized by the immune system and destroys the invading pathogen and host cell of the cancer (Finn 2005). Antibodies against tumor-associated antigen (TAA), which is present in the serum of patients with various types of cancers, can be used as a biomarker for early diagnosis of EC. These antibodies are complex and are not yet fully understood in the theory of cancer generation (Zaenker and ZimanMR 2013). Many researchers are now interested in TAA with a higher level of stability and persistence in serum samples, especially in normal human and tumor patients (Anderson and LaBaer 2005). Numerous studies have indicated that TAAs in the blood circulation of tumor patients can be detectable several years earlier than the positive imaging findings, so they can be used as novel screening markers (Tan et al. 2009). Thus, TAAs produced by autoantibodies can be biomarkers for the early detection of malignant transformation criteria for preclinical studies, which can be used as a biomarker for early detection (Tan and Zhang 2008). We will review some of the biomarkers in the serum or plasma that have been recently validated in several experiments.

Anti-p53 induced by mutant p53 protein in serum is one of the most frequently tested antibodies. With the development of molecular biotechnology, a large number of studies on the potential diagnostic value of serum p53 antibody for esophageal cancer have been published, reporting varied results. Anti-p53 is popular in various studies in China, Japan, USA, Germany, India, and other countries. The cases in these studies were mostly ESCC, and only two of them involved EAC (Kilic et al. 2008). Most antibodies have good specificity (specificity 98.3 %), but the average sensitivity is 26.7 %. Zhang et al. (2012) summarized the potential diagnostic value of serum p53 antibody in EC. Based on the current evidence, they concluded that serum p53 antibody has a potential diagnostic value for EC. However, its discrimination power is not perfect because of its low sensitivity. These results suggested that s-p53-antibody may be useful for monitoring residual tumor cells and for aiding in the selection of candidates for less invasive treatment procedures because of the high specificity of s-p53-antibody. Further studies may be needed to identify patterns of multiple biomarkers to increase the power of EC detection. A number of studies have evaluated (Xu et al. 2014) the stages at which diagnosis using autoantibodies and autoantibody panels of esophagus-specific cancer are valuable.

Shimada et al. (2005) found that four antibodies (i.e., SURF1, HOOK2, LOC146223, and AGENCOURT_7565913) are highly specific for esophageal cancer, compared with breast cancer, gastric cancer, colorectal cancer. Kilic et al. (2008) used microarrays to identify the combination of Fas ligand and anti-NY-ESO-1, and the diagnostic sensitivity and specificity obtained for the detection of EAC were 88.9 and 100 %, respectively. Autoantibody markers and a combination of antibodies and other proteins can be detected using a combination of antibody markers and conventional tumor markers, such as CEA, CYFRA211, or SCC-Ag. For example, Dong et al. (2010) found that the positive detection rate of markers for a combination of CEA, SCC, CYFRA211, and CDC25B-Abs (64.2 %) is significantly higher than that for a combination of CEA, CYFRA211, and SCC-Ag in patients with ESCC (41.0 %). In a clinical trial, Bagaria B and colleagues determined the clinical serum levels of CEA and CA199, both individually and in combination, for the diagnosis of healthy subjects and EC. Compared with the control group, serum CEA levels were significantly higher in cancer patients. The sensitivity of CEA in EC was 28 %. The sensitivity of CA19-9 was 18 %, whereas the sensitivity of CEA/CA19-9 combined in EC was 42 %. These results revealed that combined CEA and CA199 analysis indicate an increase in diagnostic sensitivity in EC (Bagaria et al. 2013).

### mRNA-based biomarkers

MicroRNA (miRNA) is a group of evolutionary conserved single chain non-coding RNAs that can participate in physiological processes, such as cell differentiation, proliferation, metabolism, and apoptosis. Some miRNAs play an important role in the tumor gene or tumor suppressor gene in the development of tumors. Circulating plasma and serum miRNAs are potential markers for noninvasive cancer diagnosis, which can be used in the diagnosis, prognosis, and targeted therapy of EC.

Increasing evidence suggested that miR-129 has the potential to become a companion diagnostic biomarker along with clinical histopathological diagnosis. In addition, miR-129 expression is reduced in patients with endometrial cancer, resulting in changes in SOX4 (sex determining region Y-box 4) in the body (Huang et al. 2009). Similar results were also found in the study of esophageal cancer and liver cancer (Kang et al. 2013). SOX4 is a transcription factor closely related to several critical pathways in the process of tumorigenesis, such as TGF-β (transforming growth factor-beta), Notch, and hedgehog signaling. It may play an important role in tumor metastasis and progression (Vervoort et al. 2013). Therefore, miR-129 may be a promising diagnostic marker for EC.

In other studies (Zhang et al. 2014), researchers have described the role of miR-200 in tumor progression, invasion, metastasis, and drug resistance. Surprisingly, miR-200b inhibits EC cell invasion in vivo without altering the expression of the two surrogate markers of the epithelial–mesenchymal transition (EMT), namely, E-cadherin and vimentin. However, whether miR-200-zeb1/2-e-cadherin cascade regulation, as a primary regulator of the EMT, is associated with the regulation of ESCC invasion remains unclear. The methylation of the E-cadherin gene is regulated by the miR-200b-zeb1/2 axis, which indicated that the e-cadherin-independent mechanism can regulate the biological function of miR-200b in ESCC. Moreover, they also found that miR-200b inhibits the integrin β1-AKT pathway via targeting kindlin-2 to reduce the invasion of ESCC. In two independent samples of EC (*n* = 20 and *n* = 53, respectively), the expression of kindlin-2 was positively correlated with the activation state of the integrin signaling pathway and the PI3 K-AKT signaling pathway (P < 0.01). These findings suggested that kindlin-2 integrin β1-AKT can function as a regulatory master in the tumor suppressor function of miR-200b in ESCC patients. In addition to our study of ESCC (Zhang et al. 2014), only one other clinical study reported the abnormal expression of miR-200 in EC, and the results showed a decrease in miR-200 expression in Barrett’s esophagus and EAC patients (Smith et al. 2011). However, information on miR-200 in ESCC remains unclear, which may be because ESCC is a malignant tumor with high frequency of local invasion and metastasis.

Various studies reported that other miRNAs are involved in the onset, development and progression of EC. Uemura and Kondo (2014) found that predictive markers for individualization of multimodality treatments are urgently needed in esophageal cancer and showed that miR-31 expression was markedly reduced in patients with poor pathological response to neoadjuvant CRT, whereas the expression of the miR-31-regulated DNA repair genes significantly increased. Luthra et al. (2008) showed that the expression of miR-196a is negatively related to A1 (ANX). ANXA1, which can mediate cell apoptosis and inhibit cell proliferation, is weakly expressed in EC tissues. Another study (Hiyoshi et al. 2009) found that miR-21 is highly expressed in EC cell lines, and it is abundantly expressed in patients with lymph node metastasis and venous invasion. Kan et al. (2009) reported that miR-106b-25 can inhibit the expression of target genes p21 and Bim and promote the transformation from Barrett to EAC. Matsushima et al. (2011) found that miR-205 can inhibit the expression of ZEB (Zinc finger E-box bindinghomeobox),thereby blocking the EMT (epithelial-to-mesenchymal transition), as well as the invasion and metastasis of ESCC. Ohta et al. (2008) reported that the tumor invasion in the low GNG7 [guanine nucleotide binding protein (G protein), gamma 7] expression group is higher than that in the high expression group; moreover, the survival rate is low, and GNG7 expression is regulated by miR-328. The low expression of miR-375 in patients with EAC is closely related to poor prognosis (Mathe et al. 2009). Furthermore, miR-27a may affect the multidrug resistance of EC; low miR-27a can significantly reduce the expression of P-glycoprotein and Bcl-2, inhibit the transcription of the multiple drug resistance-1 gene, and increase Bax expression (Zhang et al. 2010). Therefore, further research on the biological function of miRNA may provide a novel direction for the diagnosis and treatment of EC.

In addition to miRNA, long noncoding RNAs (lncRNAs), another type of RNA molecule, are steadily becoming the next frontier of cancer research. Recent findings confirmed that lncRNA is a key regulator of tumor development and progression in the esophagus. LncRNA can be easily and rapidly extracted from serum and tissue, as well as in the gastric juice in EC patients. It is expected to be a useful biomarker and therapeutic tool in clinical practice.

### Gene expression profiling biomarkers

Currently, gene expression microarray which generates quantitative expression data for thousands of genes, has been considered as a powerful tool for understanding the biological characteristics of cancers (Quackenbush 2006). In a study involving 47 patients who had a locally advanced esophageal adenocarcinoma (AC) and had undergone neoadjuvant chemotherapy with cisplatin, leucovorin and 5-fluorouracil, followed by resection. Microarray analysis was performed to discover the differential expression gene profiles. It was found that the gene encoding the ephrin B3 receptor showed the most prominent differential expression between responders and non-responders, as well as these results by immunohistochemistry (Schauer et al. 2010). Additionally, in order to identify expression patterns predictive for cisplatin-based neoadjuvant chemotherapy, Motoori et al. (2010) analysed comprehensive gene expression profiling of pretreatment biopsy tissues from 25 patients with esophageal squamous cell carcinoma (SCC). Their system consisted of 199 most informative genes and had the prediction accuracy of 82 %. Duong et al. (2007) performed microarray analysis for 46 esophageal cancer patients, that is, 21 SCC and 25 AC patients for whom neoadjuvant CRT had been recommended. Their study was based on two-color competitive hybridization to a cDNA array printed at the Peter MacCallum Cancer Centre Microarray Core Facility and identified a 32-gene classifier that could be used to predict a response to neoadjuvant CRT in SCCs, whereas a negative predictive profile was observed for AC patients. These examples suggest that gene expression profiling is a powerful tool to identify gene sets for selection of optimal and personalized therapy for patients with esophageal cancer.

## Conclusion

Cancer biomarkers have provided some promising therapeutic targets for the diagnosis, treatment, and prognosis of EC. They are needed to develop a treatment strategy for EC, which can be integrated with diverse clinical characteristics. The current study has shed light on the limitations of EC biomarkers, and further technological advances can be anticipated. For example, analytical methods, such as blood test, IHC, molecular pathology, gene expression profile and biopsy, will be helpful in finding the appropriate antibodies to discover and verify more biomarker candidates. To obtain research data, in addition to improving the performance of the biomarkers, interdisciplinary cooperation is necessary. These results can benefit patients with EC, as well as other cancer patients.

## References

[CR1] Akamatsu M, Matsumoto T, Oka K, Yamasaki S, Sonoue H, Kajiyama Y (2003). c-erbB-2 oncoprotein expression related to chemoradioresistance in esophageal squamous cell carcinoma. Int J Radiat Oncol Biol Phys.

[CR2] Anderson KS, LaBaer J (2005). The sentinel within: exploiting the immune system for cancer biomarkers. J Proteome Res.

[CR3] Bagaria B, Sood S, Sharma R, Lalwani S (2013). Comparative study of CEA and CA19-9 in esophageal, gastric and colon cancers individually and in combination (ROC curve analysis). Cancer Biol Med.

[CR4] Berry MF (2014). Esophageal cancer: staging system and guidelines for staging and treatment. J Thorac Dis.

[CR5] Bird-Lieberman EL, Dunn JM, Coleman HG, Lao-Sirieix P, Oukrif D, Moore CE (2012). Population-based study reveals new risk-stratification biomarker panel for Barrett’s esophagus. Gastroenterology.

[CR6] Cerfolio RJ, Bryant AS, Ohja B, Bartolucci AA, Eloubeidi MA (2005). The accuracy of endoscopic ultrasonography with fine-needle aspiration, integrated positron emission tomography with computed tomography, and computed tomography in restaging patients with esophageal cancer after neoadjuvant chemoradiotherapy. J Thorac Cardiovasc Surg.

[CR7] Chan DS, Twine CP, Lewis WG (2012). Systematic review and meta-analysis of the influence of HER2 expression and amplification in operable oesophageal cancer. J Gastrointest Surg.

[CR8] DiMaio MA, Kwok S, Montgomery KD, Lowe AW, Pai RK (2012). Immunohistochemical panel for distinguishing esophageal adenocarcinoma from squamous cell carcinoma: a combination of p63, cytokeratin 5/6, MUC5AC, and anterior gradient homolog 2 allows optimal subtyping. Hum Pathol.

[CR9] Dong J, Zeng BH, Xu LH, Wang JY, Li MZ, Zeng MS (2010). Anti-CDC25B autoantibody predicts poor prognosis in patients with advanced esophageal squamous cell carcinoma. J Transl Med.

[CR10] Dreilich M, Wanders A, Brattstrom D, Bergstrom S, Hesselius P, Wagenius G (2006). HER-2 overexpression (3+) in patients with squamous cell esophageal carcinoma correlates with poorer survival. Dis Esophagus.

[CR11] Duong C, Greenawalt DM, Kowalczyk A, Ciavarella ML, Raskutti G, Murray WK (2007). Pretreatment gene expression profiles can be used to predict response to neoadjuvant chemoradiotherapy in esophageal cancer. Ann Surg Oncol.

[CR12] Enzinger PC, Mayer RJ (2003). Esophageal cancer. N Engl J Med.

[CR13] Finn OJ (2005). Immune response as a biomarker for cancer detection and a lot more. N Engl J Med.

[CR14] Fitzgerald RC, di Pietro M, Ragunath K, Ang Y, Kang JY, Watson P (2014). British Society of Gastroenterology guidelines on the diagnosis and management of Barrett’s oesophagus. Gut.

[CR15] Glickman JN, Yang A, Shahsafaei A, McKeon F, Odze RD (2001). Expression of p53-related protein p63 in the gastrointestinal tract and in esophageal metaplastic and neoplastic disorders. Hum Pathol.

[CR16] Hiyoshi Y, Kamohara H, Karashima R, Sato N, Imamura Y, Nagai Y (2009). MicroRNA-21 regulates the proliferation and invasion in esophageal squamous cell carcinoma. Clin Cancer Res.

[CR17] Huang YW, Liu JC, Deatherage DE, Luo J, Mutch DG, Goodfellow PJ (2009). Epigenetic repression of microRNA-129-2 leads to overexpression of SOX4 oncogene in endometrial cancer. Cancer Res.

[CR18] Jemal A, Bray F, Center MM, Ferlay J, Ward E, Forman D (2011). Global cancer statistics. CA Cancer J Clin.

[CR19] Kan T, Sato F, Ito T, Matsumura N, David S, Cheng Y (2009). The miR-106b-25 polycistron, activated by genomic amplification, functions as an oncogene by suppressing p21 and Bim. Gastroenterology.

[CR20] Kang M, Li Y, Liu W, Wang R, Tang A, Hao H (2013). miR-129-2 suppresses proliferation and migration of esophageal carcinoma cells through downregulation of SOX4 expression. Int J Mol Med.

[CR21] Kaye PV, Haider SA, Ilyas M, James PD, Soomro I, Faisal W (2009). Barrett’s dysplasia and the Vienna classification: reproducibility, prediction of progression and impact of consensus reporting and p53 immunohistochemistry. Histopathology.

[CR22] Kilic A, Schuchert MJ, Luketich JD, Landreneau RJ, Lokshin AE, Bigbee WL (2008). Use of novel autoantibody and cancer-related protein arrays for the detection of esophageal adenocarcinoma in serum. J Thorac Cardiovasc Surg.

[CR23] Kumar A, Chatopadhyay T, Raziuddin M, Ralhan R (2007). Discovery of deregulation of zinc homeostasis and its associated genes in esophageal squamous cell carcinoma using cDNA microarray. Int J Cancer.

[CR24] Lesnikova I, Lidang M, Hamilton-Dutoit S, Koch J (2009). HER2/neu (c-erbB-2) gene amplification and protein expression are rare in uterine cervical neoplasia: a tissue microarray study of 814 archival specimens. APMIS.

[CR25] Luthra R, Singh RR, Luthra MG, Li YX, Hannah C, Romans AM (2008). MicroRNA-196a targets annexin A1: a microRNA-mediated mechanism of annexin A1 downregulation in cancers. Oncogene.

[CR26] Mathe EA, Nguyen GH, Bowman ED, Zhao Y, Budhu A, Schetter AJ (2009). MicroRNA expression in squamous cell carcinoma and adenocarcinoma of the esophagus: associations with survival. Clin Cancer Res.

[CR27] Matsushima K, Isomoto H, Yamaguchi N, Inoue N, Machida H, Nakayama T (2011). MiRNA-205 modulates cellular invasion and migration via regulating zinc finger E-box binding homeobox 2 expression in esophageal squamous cell carcinoma cells. J Transl Med.

[CR28] Motoori M, Takemasa I, Yamasaki M, Komori T, Takeno A, Miyata H (2010). Prediction of the response to chemotherapy in advanced esophageal cancer by gene expression profiling of biopsy samples. Int J Oncol.

[CR29] Ohta M, Mimori K, Fukuyoshi Y, Kita Y, Motoyama K, Yamashita K (2008). Clinical significance of the reduced expression of G protein gamma 7 (GNG7) in oesophageal cancer. Br J Cancer.

[CR30] Pacha A, Rygiel AM, Westra W, Dijkgraaf MG, Rosmolen W, Visser M (2012). Su1181 A diagnostic DNA fish biomarker assay identifies HGD or EAC in Barrett esophagus. Elsevier Gastroenterol.

[CR31] Penault-Llorca F, Bilous M, Dowsett M, Hanna W, Osamura RY, Ruschoff J (2009). Emerging technologies for assessing HER2 amplification. Am J Clin Pathol.

[CR32] Quackenbush J (2006). Microarray analysis and tumor classification. N Engl J Med.

[CR33] Rice TW, Zuccaro G, Adelstein DJ, Rybicki LA, Blackstone EH, Goldblum JR (1998). Esophageal carcinoma: depth of tumor invasion is predictive of regional lymph node status. Ann Thorac Surg.

[CR34] Rice TW, Rusch VW, Apperson-Hansen C, Allen MS, Chen LQ, Hunter JG (2009). Worldwide esophageal cancer collaboration. Dis Esophagus.

[CR35] Rygiel AM, van Baal JWPM, Milano F, Wang KK, ten Kate FJ, Fockens P (2007). Efficient automated assessment of genetic abnormalities detected by fluorescence in situ hybridization on brush cytology in a Barrett esophagus surveillance population. Cancer.

[CR36] Salehi M, Moradi-Lakeh M, Salehi MH, Nojomi M, Kolahdooz F (2013). Meat, fish, and esophageal cancer risk: a systematic review and dose-response meta-analysis. Nutr Rev.

[CR37] Schauer M, Janssen KP, Rimkus C, Raggi M, Feith M, Friess H (2010). Microarray-based response prediction in esophageal adenocarcinoma. Clin Cancer Res.

[CR38] Shimada H, Nakashima K, Ochiai T, Nabeya Y, Takiguchi M, Nomura F (2005). Serological identification of tumor antigens of esophageal squamous cell carcinoma. Int J Oncol.

[CR39] Sikkema M, Kerkhof M, Steyerberg EW, Kusters JG, van Strien PMH, Looman CWN (2009). Aneuploidy and overexpression of Ki67 and p53 as markers for neoplastic progression in Barrett’s esophagus: a case–control study. Am J Gastroenterol.

[CR40] Skacel M, Petras RE, Rybicki LA, Gramlich TL, Richter JE, Falk GW (2002). p53 expression in low grade dysplasia in Barrett’s esophagus: correlation with interobserver agreement and disease progression. Am J Gastroenterol.

[CR41] Smith CM, Watson DI, Leong MP, Mayne GC, Michael MZ, Wijnhoven BP (2011). miR-200 family expression is downregulated upon neoplastic progression of Barrett’s esophagus. World J Gastroenterol.

[CR42] Spector NL, Blackwell KL (2009). Understanding the mechanisms behind trastuzumab therapy for human epidermal growth factor receptor 2-positive breast cancer. J Clin Oncol.

[CR43] Sudo T, Mimori K, Nagahara H, Utsunomiya T, Fujita H, Tanaka Y, Shirouzu K, Inoue H, Mori M (2007). Identification of EGFR mutations in esophageal cancer. Eur J Surg Oncol.

[CR44] Tan EM, Zhang J (2008). Autoantibodies to tumor-associated antigens: reporters from the immune system. Immunol Rev.

[CR45] Tan HT, Low J, Lim SG, Chung M (2009). Serum autoantibodies as biomarkers for early cancer detection. FEBS J.

[CR46] Thompson SK, Ruszkiewicz AR, Jamieson GG, Esterman A, Watson DI, Wijnhoven BP (2008). Improving the accuracy of TNM staging in esophageal cancer: a pathological review of resected specimens. Ann Surg Oncol.

[CR47] Uemura N, Kondo T (2014). Current status of predictive biomarkers for neoadjuvant therapy in esophageal cancer. World J Gastrointest Pathophysiol.

[CR48] Vervoort SJ, van Boxtel R, Coffer PJ (2013). The role of SRY-related HMG box transcription factor 4 (SOX4) in tumorigenesis and metastasis: friend or foe?. Oncogene.

[CR49] Wang KL, Wu TT, Choi IS, Wang H, Resetkova E, Correa AM (2007). Expression of epidermal growth factor receptor in esophageal and esophagogastric junction adenocarcinomas: association with poor outcome. Cancer.

[CR50] Wang Q, Zhu H, Xiao Z, Zhang W, Liu X, Zhang X (2013). Expression of epidermal growth factor receptor is an independent prognostic factor for esophageal squamous cell carcinoma. World J Surg Oncol.

[CR51] Weston AP, Banerjee SK, Sharma P, Tran TM, Richards R, Cherian R (2001). p53 protein overexpression in low grade dysplasia (LGD) in Barrett’s esophagus: immunohistochemical marker predictive of progression. Am J Gastroenterol.

[CR52] Xu YW, Peng YH, Chen B, Wu ZY, Wu JY, Shen JH (2014). Autoantibodies as potential biomarkers for the early detection of esophageal squamous cell carcinoma. Am J Gastroenterol.

[CR53] Zaenker P, Ziman MR (2013). Serologic autoantibodies as diagnostic cancer biomarkers—a review. Cancer Epidemiol Biomarkers Prev.

[CR54] Zali H, Ahmadi G, Bakhshandeh R, Rezaei-Tavirani M (2011). Proteomic analysis of gene expression during human esophagus cancer. J Paramed Sci.

[CR55] Zhang H, Li M, Han Y, Hong L, Gong T, Sun L (2010). Down-regulation of miR-27a might reverse multidrug resistance of esophageal squamous cell carcinoma. Dig Dis Sci.

[CR56] Zhang J, Xv Z, Wu X, Li K (2012). Potential diagnostic value of serum p53 antibody for detecting esophageal cancer: a meta-analysis. PLoS ONE.

[CR57] Zhang HF, Xu LY, Li EM (2014). A family of pleiotropically acting microRNAs in cancer progression, miR-200: potential cancer therapeutic targets. Curr Pharm Des.

[CR58] Zhang HF, Zhang K, Liao LD, Li LY, Du ZP, Wu BL (2014). miR-200b suppresses invasiveness and modulates the cytoskeletal and adhesive machinery in esophageal squamous cell carcinoma cells via targeting Kindlin-2. Carcinogenesis.

[CR59] Zuguchi M, Miki Y, Onodera Y, Fujishima F, Takeyama D, Okamoto H (2012). Estrogen receptor alpha and beta in esophageal squamous cell carcinoma. Cancer Sci.

